# The Chinese Herbal Formula PAPZ Ameliorates Behavioral Abnormalities in Depressive Mice

**DOI:** 10.3390/nu11040859

**Published:** 2019-04-16

**Authors:** Huiling Chen, Qing Huang, Shunjia Zhang, Kaiqiang Hu, Wenxiang Xiong, Lingyun Xiao, Renhuai Cong, Qingfei Liu, Zhao Wang

**Affiliations:** 1 MOE Key Laboratory of Protein Science, School of Pharmaceutical Sciences, Tsinghua University, Beijing 100084, China; chenhl16@mails.tsinghua.edu.cn (H.C.); qinghuangfgh@163.com (Q.H.); shunjia.zhang@yale.edu (S.Z.); hkq15@mails.tsinghua.edu.cn (K.H.); xiongwxiang@163.com (W.X.); 2 School of Life Science and Technology, Tokyo Institute of Technology, Yokohama 2668501, Japan; 3 Environmental Health Science, School of Public Health, Yale University, New Haven, CT 06520, USA; 4 Research & Development Centre, Infinitus (China) Company Ltd., Guangzhou 510663, China; xiaolingyun12@126.com (L.X.); Renhuai.Cong@infinitus-int.com (R.C.)

**Keywords:** major depressive disorder, Chinese herbal formula, corticosterone, BDNF, oxidative stress

## Abstract

Major depressive disorder (MDD) is a chronic mental disorder characterized by mixed symptoms and complex pathogenesis. With long history of practical application, traditional Chinese medicine (TCM) offers many herbs for the treatment and rehabilitation of chronic disease. In this study, we developed a modified Chinese herbal formula using *Panax ginseng, Angelica Sinensis, Polygala tenuifolia* Willd, and *Ziziphi spinosae* Semen (PAPZ), based on an ancient TCM prescription. The antidepressant effects of PAPZ were investigated with a corticosterone (CORT) model of depression in mice. Our results showed that administration of PAPZ ameliorated depression-like phenotypes in the CORT model. An anatomic study showed that chronic PAPZ administration upregulated the protein expression of brain-derived neurotrophic factor (BDNF) in hippocampal tissue. The enzyme activity of superoxide dismutase was enhanced in hippocampal tissue, in line with a decreased malondialdehyde level. Taken together, these findings suggested that PAPZ has therapeutic effects in a mice depression model through increasing protein expression of BDNF and improving the anti-oxidation ability of the brain.

## 1. Introduction

Major depressive disorder (MDD) or depression, which has an estimated global prevalence of 4.7%, is a mental disorder that affects human thoughts, mood, and physical health. It can occur at as early as 3 years of age, and appears across all world regions [1,2]. The global burden of disease (GBD) in 2010 identified MDD as a leading contributor to the global disease burden [3]. Similar to anxiety disorder, MDD has been reported to cause brain injury, especially damaging the hippocampus region of the brain, causing neuronal dysfunction or affecting neural plasticity [4]. Brain-derived neurotrophic factor (BDNF) plays a critical neurotrophic role in neural plasticity [5,6]. In prior studies, *bdnf* has been identified as a target gene in depression treatment [5,7,8]. Adachi et al. [9] reported that the loss of BDNF in the hippocampal tissue contributed to increasing vulnerability to depression, whereas upregulation mediated antidepressant efficacy.

MDD has been thought to be a heterogeneous disease with diverse etiological and multifactorial pathogenesis [10,11]. Modern medicine has primarily focused on symptomatic treatment, and drugs are mainly applied in single-target and single-factor therapy. Traditional Chinese medicine (TCM), whose treatments are designed systematically based upon the constitution of patients, has a long history and the potential to treat many diseases including depressive-like syndromes [10,12]. Chinese herbal medicine is the major form of prescription of TCM. Chinese herbal formulas (CHFs), which aim to help patients re-achieve the Yin–Yang balance of the body, usually consist of multiple herbs that synchronize with one other when administered. Considerable progress has occurred not only in basic research, but also in clinical research, with a better understanding of the underlying neurobiological basis of CHFs. It is believed that a multi-component and multi-target mechanism may be the essential mechanism through which CHF achieve holistic effects [13].

The most common traditional method of preparing CHFs is through the water decocting method. In brief, prepared slices of medical herbs are boiled in hot water, and the decoction administrated orally [14]. Aiming to seek CHFs that have the potential to serve as alternatives for MDD treatment, Infinitus (China) Co., Ltd. offered 22 modified CHFs for regulating mood. We then explored whether the decoction of them had protective effects on hippocampal neuronal cell line (HT22) against corticosterone (CORT)-induced apoptosis (Supplementary Table S1). We found that the formula WXJ-17-001, which comprises *Panax ginseng, Angelica ginensis, Polygala tenuifolia* Willd, and *Ziziphi spinosae* Semen, had the best protection effect among them. We then renamed the formula according to its composing as PAPZ.

Each single herb of PAPZ is beneficial for central nervous system disorders and widely used in an Asian-medicated diet. *P. ginseng* is a widely-used medicinal plant in Asian countries; it has various pharmacological effects such as neuroprotection, endocrine modulation, cardiovascular protection, and immunoregulation [10]. Many research articles have reported that *P. ginseng* or its components are beneficial to the central nervous system, and have therapeutic effects on neuropsychiatric disorders [15]. *A. sinensis*, known as *Dong-Gui*, is served as a healthy food and is also a common traditional medicine used for the treatment of cerebrovascular diseases [16,17]. A study showed that the formula *Dang-Gui-Shao-Yao-San*, of which the major active component is *A. sinensis*, had pharmaceutical effects on depression [18]. *P. tenuifolia* Willd is used as an expectorant, tonic, tranquillizer, or antipsychotic agent in TCM [19]. *Senegenin*, extracted from *P. tenuifolia* Willd, shows an antidepressant effect by inhibiting the nuclear factor κ-light-chain-enhancer of activated B cells (NF-κB) pathway [20]. *Z. spinosae* Semen is the mature seed of sour jujube, and is used as a sedative agent in TCM [21,22,23]. *Z. spinosae* Semen could improve learning and memory of mice [24]. It has been reported to have an antidepressant effect in a rat depressive model [25]. However, whether a formula of these herbs would have antidepressant effects, and the underlying molecular mechanism, remains unclear.

In the present study, chronic treatment with corticosterone (CORT), which is a common inducer of depression-related behavior both in rats and mice [26,27], was used to establish a depressive-like mice model. A hippocampus-derived HT22 cell line was applied to in vitro experiments. Our results showed that administration of PAPZ ameliorated depressive-like phenotypes by improving the protein expression of BDNF and the activity of superoxide dismutase (SOD) of hippocampal tissue in mice. PAPZ also attenuated CORT-induced apoptosis in HT22 cell in vitro. Taken together, the results demonstrated the neuroprotective effect of PAPZ, which suggested PAPZ is a good candidate for treatment of depressive disorders.

## 2. Materials and Methods

### 2.1. Materials

CORT was purchased from Takyo Chemical Industry Co., Ltd. (Shanghai, China). The Chinese herbs *P. ginseng*, *A. sinensis*, *P. tenuifolia* Willd, and *Z. spinosae* Semen were purchased from Beijing Tong Ren Tang Guangzhou Pharmaceutical chain Co., Ltd. (Guangzhou, China). The HT22 cell line was purchased from Tong Pai Biotechnology Co., Ltd. (Shanghai, China). Dulbecco’s Modified Eagle Medium (DMEM) and penicillin/streptomycin were obtained from Corning Incorporated (Corning, NY, USA). Fetal bovine serum (FBS) was purchased from Invitrogen Corporation (Carlsbad, CA, USA). The cell counting kit-8 (CCK-8) and fluorescein isothiocyanate (FITC)-Annexin V apoptosis detection kit were purchased from Beyotime Biotechnology (Shanghai, China). Real-time quantitative polymerase chain reaction (qPCR)-related kits, the FastQuant RT kit, and Super Real PreMix Plus were purchased from Tiangen Biotech CO., Ltd. (Beijing, China). The SOD assay kit and malondialdehyde (MDA) assay kit were obtained from Nanjing Jiancheng Biotechnology Co., Ltd. (Nanjing, China). Anti-BDNF and anti-β-tubulin were purchased from Cell Signaling Technology (Danvers, MA, USA). All other chemicals were of analytical grade and used as received.

### 2.2. Preparation of CHFs and CORT

All CHFs used in this study were prepared using the water decocting method from dried medical herbs purchased in the form of prepared slices. Water was added to the mixture of prepared slices of the herbs in a 1:10 (g/mL) ratio of materials to liquid and boiled for 2 h. The decoction was collected and the same amount of water was added to the residue. Then, the mixture was boiled for another 2 h. Next, the decoctions were combined and concentrated to a constant volume. The water extract was centrifuged (13,000 rpm, 10 min, 4 °C) to separate any insoluble materials, and then the supernatant was filtered through a 0.2 μm syringe filter before use. The prepared water extract was frozen at −20 °C for storage. Four Chinese medicine herbs, *P. ginseng*, *A. sinensis*, *P. tenuifolia* Willd, and *Z. spinosae* Semen, were included in PAPZ (Table 1). The dosage of PAPZ described in this paper was in the form of equivalent dry herb amount.

Corticosterone (CORT) powder was suspended in 0.9% (w/v) physiological saline with 0.1% dimethyl sulfoxide (DMSO) acting as a cosolvent.

### 2.3. Animals and Treatment

Male C57BL/6J mice weighing 18–22 g were obtained from the laboratory animal center of Tsinghua University (Beijing, China) and were housed in cages with free access to food and water in a room with an ambient temperature of 22 ± 2 °C and a 12 h light/dark cycle. All animal experiments were conducted according to the relevant guidelines and regulations and with the approval of the Institutional Ethical Committee of China.

After an adaptive phase of 3 days, the mice were randomly divided into the control, CORT, and PAPZ+CORT group (*n* = 10/group). The dose of CORT (40 mg/kg) was selected based on data from the literature [28,29] and PAPZ (1000 mg/kg) treatment was calculated by extrapolating the human recommended daily dosage of the single herb according to the Chinese Pharmacopoeia. For the control group and CORT group, normal saline was administrated to mice by oral gavage first, and 30 min later, the mice were subcutaneously injected with saline or CORT. For the PAPZ+CORT group, the mice were administrated with PAPZ by oral gavage 30 min prior to CORT supplement. All drugs were administrated at a volume of 10 mL/kg. During behavioral assessments, the administration of all drugs continued.

### 2.4. Open Field Test

An open field test (OFT) was conducted 24 h after the 21 days of CORT treatment. An open box (60 × 60 × 50 cm) was used in the OFT. At the beginning of the test period, mice were placed in the center of the box to adapt for 3 min. The experimental parameters were set, and the test time was set to 10 min. The locomotor activity (movement distance, movement speed, and other indicators) was monitored and traced with an automated video-tracking system. Between one mouse and the next one, the apparatus was thoroughly cleaned with 75% ethanol, followed by distilled water and dried before using.

### 2.5. Novel Object Recognition Test

A novel object recognition (NOR) test was conducted after OFT. NOR is a commonly used behavioral assay for the investigation of various aspects of learning and memory level in mice. NOR is fairly simple and can be completed over 5 days: habituation (3 days), training (day 4), and testing day (day 5). During training, the mouse was allowed to explore two identical objects in the open field area (50 × 50 cm). On testing day, one of the training objects was replaced with a novel object. The exploring time of the novel object and familiar object was recorded within 5 min. The discrimination index (DI) was used to evaluate the learning and memory ability of animals. The DI calculation formula is as follows:

DI = (N – F) / (N + F) × 100%,

where N is the time spent on novel location/object exploration and F is the time spent on familiar location/object exploration.

### 2.6. Tail Suspension Test

A tail suspension test (TST) was conducted after NOR. Briefly, mice were individually suspended 5 cm above the bottom of the TST box. The tip of the tail was stuck with adhesive tape. Immobility time was detected during the last 4 min of total 6 min test.

### 2.7. Morris Water Maze Experiment

A Morris water maze (MWM) experiment was conducted in submerged platform following standard procedures and it was conducted after TST. The hidden-platform was fixed in the center of one of quadrants and 1.5 cm below the water surface. Mice underwent three trials each day for 6 days. Each time, mice were placed into the water from other three quadrants. Before and after each test, mice were placed on the platform to adapt for 20 s. Mice that remained on the platform for more than 5 s were considered to have searched platform successfully during the 90 s of detection. The mice were deeply anesthetized and sacrificed by cervical dislocation after MWM test was finished.

Once the mice were sacrificed, the hippocampal tissues were quickly dissected and immersed immediately in liquid nitrogen and then transferred to refrigerator at −80 °C until being used.

### 2.8. Real-time Quantitative PCR and Western Blotting

For qPCR analysis, total RNA from hippocampal tissues were extracted using trizol regent according to the manufacturer’s instructions. Complementary deoxyribonucleic acid (cDNA) was obtained by using a FastQuant RT Kit and real-time PCR analysis was used with SuperReal PreMix Plus according to the 2-step reaction program. qPCR analysis was conducted using primers as follows: *gapdh* forward 5′-CATGGCCTTCCGTGTTCCTA-3′, reverse 5′-CCTGCTTCACCACCTTCTTGAT-3′, *bdnf* forward 5′-GCCTTCATGCAACCGAAGTA-3′, reverse 5′-TGAGTCTCCAGGACAGCAAA-3′.

For immunoblot analysis, hippocampal tissues were resuspended with lysis buffer. The protein was extracted and samples were resolved on 10% sodium dodecyl sulfonate–polyacrylamide gel (SDS-PAGE) and then transferred to polyvinylidene fluoride (PVDF) membranes. Antibodies against the following proteins were used: BDNF and β-tubulin goat anti-rabbit immunoglobulin G (IgG).

### 2.9. SOD and MDA Assay

The supernatant of hippocampal tissues was used to detect SOD activity and MDA level using SOD assay kit and MDA assay kit, respectively, following the manufacturer’s instructions.

### 2.10. Determination of Cell Viability

HT22 cells were seeded into 10-cm dishes and cultured in DMEM supplemented with 10% FBS and 1% penicillin-streptomycin at 37 °C in a humidified incubator containing 5% CO_2_.

Cell viability was measured by CCK-8 assay. Briefly, the cells were cultured at a density of 5 × 10^3^ cells per well into 96-well plates. When the cells reached 70–80% confluence, they were pre-treated with 400 µM CORT for 24 h, and then treated with PAPZ with concentrations of 0.5, 5.0, and 10.0 mg/mL for 24 h in the presence of CORT. In this study, 400 μM CORT was determined as the optimal dose of cell damage model through preliminary experiments (Supplementary Figure S1). The absorbance at 450 nm was measured in an enzyme standard instrument.

### 2.11. Determination of Cell Apoptosis by Flow Cytometry

The apoptotic cells were measured using the FITC-Annexin V Apoptosis Detection Kit. After drug treatment as a cell viability experiment, cells were harvested and then washed with 1 mL PBS. Next, cells were resuspended in 195 μL FITC-Annexin V binding buffer, and then 5 µL of FITC-Annexin V and 10 µL propidium iodide (PI) incubation buffer were added according to the manufacturer’s instructions. The cells were incubated for 15 min at room temperature and protected from light. Samples were not stored, but analyzed immediately. Samples were analyzed with an imaging flow cytometer.

### 2.12. Statistical Analysis

Results are presented as mean ± SEM. For the in vitro experiments, all results reported were formed at least three independent experiments (*n* = 6 for each group). For the in vivo animal experiments, each group contained 10 mice. Data were analyzed with Graph-Pad Prism 5.0 software (GraphPad Software Inc., San Diego, CA, USA). Two-tailed, unpaired *t*-tests were used to compare two groups. Differences were considered significant when *p* < 0.05. To directly compare the effect of PAPZ treatment, data from the CORT group and PAPZ+CORT group were normalized to the control group.

## 3. Results

### 3.1. Antidepressant Effects of PAPZ

**Figure 1:**
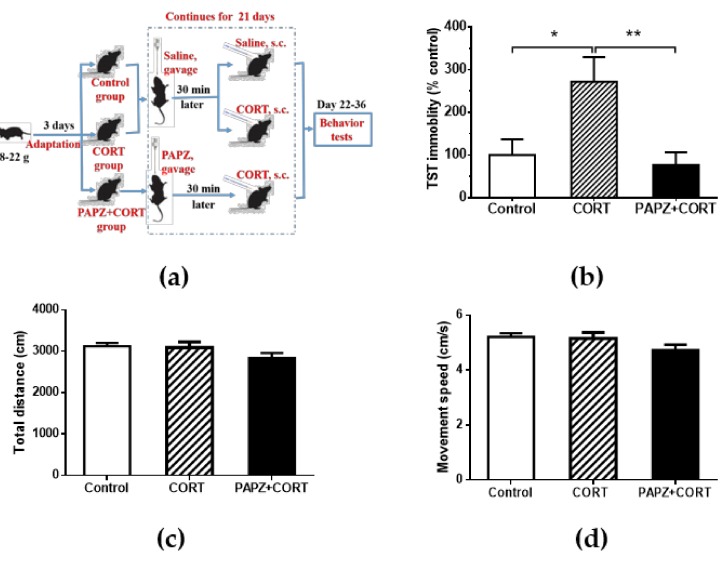
PAPZ ameliorated depressive-like behavior induced by corticosterone (CORT) in mice. (**a**) Schematic representation of the treatment protocol. (**b**) The immobility time in the tail suspension test (TST). (**c**) Total distance travelled in the open field test (OFT). (**d**) Movement speed during bouts of walking in the OFT. Data from the CORT group and PAPZ+CORT group were normalized to the control group and data are expressed as mean ± SEM. * *p* < 0.05, ** *p* < 0.01 represent significant differences. s.c.: sub-cutaneous.

To investigate the effect of PAPZ on depressive-like behavior, we chose the chronic CORT model of depression, which has been widely used in depression-related research (Figure 1). Mice were administrated subcutaneously with CORT once daily for 21 consecutive days, and PAPZ were administered by oral gavage 30 minutes prior to the CORT injection. Behavioral tests were performed after the treatments had finished (Figure 1a). The chronic CORT injection resulted in a significant prolonged immobility time in the TST when compared with the control group (Figure 1b). The PAPZ+CORT treated group exhibited a significantly decreased immobility time compared with the model group, which suggested that PAPZ could protect against the CORT-induced depressive-like behavior in the TST test. An OFT was conducted to assess the influence of PAPZ on the locomotor activities of mice. PAPZ administration had no significant impact on the total distance and movement speed of mice spent in the zone in the OFT (Figure 1c,d).

### 3.2. Effects of PAPZ on Learning and Memory Capacity

**Figure 2:**
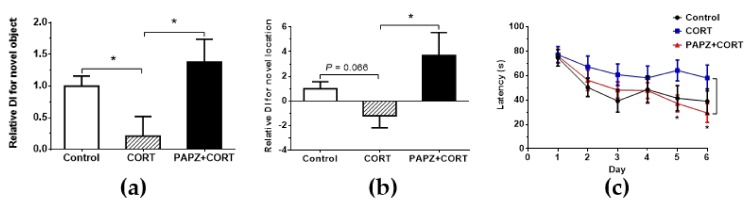
PAPZ ameliorated learning and memory impairment induced by CORT in mice. (**a**,**b**) The relative discrimination index (DI) for the novel object, (**a**) for novel location and (**b**) in the novel object recognition (NOR) test. (**c**) The total latency in the Morris water maze (MWM) test. Data from the CORT group and PAPZ+CORT group were normalized to the control group and data are expressed as mean ± SEM. * *p* < 0.05 represents a significant difference.

We evaluated the effect of PAPZ on cognitive capacity in CORT-induced mice using the NOR test and learning, and memory ability using the MWM test, respectively (Figure 2). The discrimination index (DI), representing cognitive ability, was determined from the NOR task. CORT induced a significant decrease in the DI score for the novel object when compared with the control group. As expected, we found a significant increase in the DI score for the novel object in the PAPZ+CORT treated group when compared to the group treated with CORT alone. The DI score for the novel location in the CORT-treated group demonstrated a decreasing tendency, although there was no significant difference when compared with the control group (Figure 2a). Treatment with PAPZ significantly increased the DI score for the novel location when compared with the CORT group (Figure 2b). In the MWM test, latency refers to the time spent by the mouse to find the platform. There was a relative increasing tendency of latency in the chronic CORT-treated group when compared with the control group. PAZA treatment significantly decreased the latency when compared with the CORT-treated group, especially on the fifth and sixth day (Figure 2c). All these results indicated that PAPZ could ameliorate depressive-like behavior and improve cognitive capacity and learning ability in depressive-like mice induced by the chronic administration of CORT.

### 3.3. Molecular Mechanism of the Cerebral Protection Effect of PAPZ

**Figure 3:**
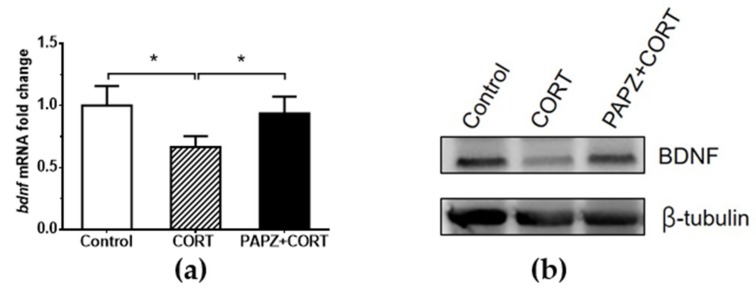
PAPZ enhanced the expression of brain-derived neurotrophic factor BDNF in hippocampal tissue. (**a**) Real-time quantitative polymerase chain reaction (qPCR) analysis of *bdnf* gene in the hippocampal tissues in mice. (**b**) Representative Western blots show the difference of BDNF protein expression in hippocampal tissue. Data from the CORT group and PAPZ+CORT group were normalized to the control group and data are expressed as mean ± SEM. * *p* < 0.05 represents a significant difference.

To investigate the underlying molecular mechanism of PAPZ in CORT-induced depressive-like mice, we examined the effect of PAPZ on the expression of *bdnf* on hippocampal tissue in mice (Figure 3). The real-time qPCR result demonstrated that the mRNA level of *bdnf* significantly decreased by almost 50% when compared with the non-treated control group. Treatment with PAPZ showed that the *bdnf* values were significantly increased when compared with the CORT group, which indicated that the PAPZ could promote the expression of *bdnf* at the level of transcription (Figure 3a). We next detected the expression of BDNF protein. The immunoblot result was consistent with the qPCR result (Figure 3b) and revealed that the expression of BDNF protein in the model group significantly decreased after treatment with CORT, and PAPZ could enhance the expression of BDNF protein, which indicates that PAPZ could ameliorate the damage of CORT to hippocampus tissues.

### 3.4. PAPZ Enhanced Cerebral Antioxidant Ability of Tested Mice

**Figure 4:**
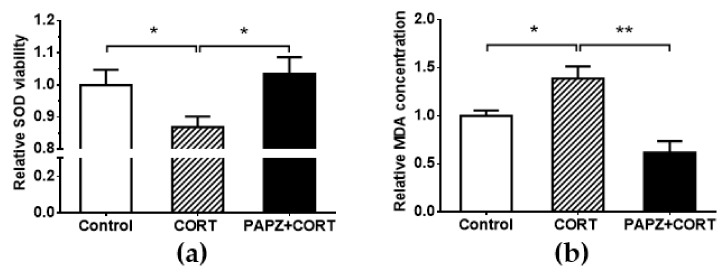
PAPZ enhanced superoxide dismutase (SOD) viability and decreased malondialdehyde (MDA) level in depressive-like mice. (**a**) The SOD activity of hippocampal tissue in mice was measured by the SOD assay kit. (**b**) The MDA activity of hippocampal tissue in mice was measured by the MDA assay kit. Data from the CORT group and PAPZ+CORT group were normalized to the control group and data are expressed as mean ± SEM. * *p* < 0.05, ** *p* < 0.01 represent significant differences.

Oxidative stress has been shown to cause neuronal degeneration and to play a role in the pathogenesis of anxiety and depression [30,31]. We hypothesized that PAPZ exerted neuroprotective effects by reducing cerebral oxidative stress and verified the hypothesis by SOD and MDA assays (Figure 4). Reactive oxygen species (ROS) are a kind of free radical that can damage cells through enzyme inactivation, lipid peroxidation, DNA modification, and other pathways [32]. Studies have reported that SOD is an important endogenous antioxidant enzyme and an important part of the first-line defense system against ROS [33,34]. Therefore, the SOD viability of hippocampal tissue was detected in mice. CORT treatment significantly deceased the SOD viability when compared with the control group, and PAPZ treatment significantly increased the SOD viability when compared with the CORT-treated group (Figure 4a). These results indicate that PAPZ could improve the SOD viability of mice in vivo.

MDA is a metabolite of lipid peroxidation, and its content can indirectly reflect the degree of damage of lipid peroxidation. Studies have reported that MDA concentrations in depressed patients increased when compared with healthy control groups [35,36]. The concentration of MDA in the hippocampal tissue in the CORT group significantly increased after treatment with CORT when compared with that in the control group. The change in MDA content in the PAPZ+CORT group was, as opposed to SOD, markedly decreased when compared with that in the CORT group (Figure 4b). All the results indicated that PAPZ could enhance the cerebral antioxidant ability in mice treated with CORT.

### 3.5. PAPZ Protected Neurons In Vitro

**Figure 5:**
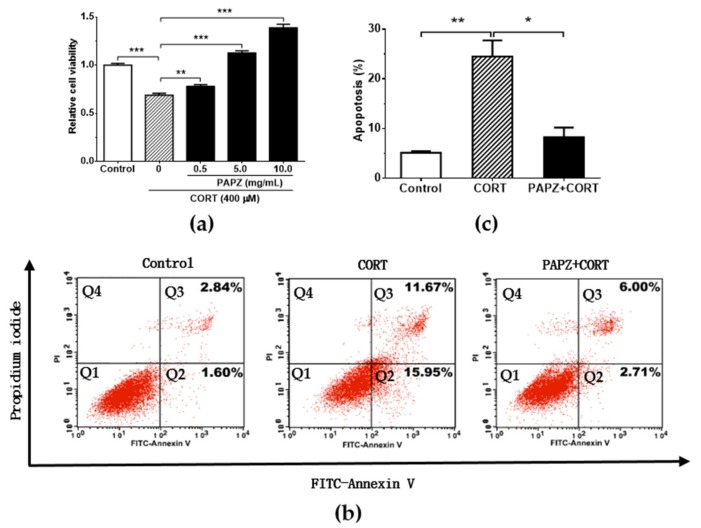
PAPZ attenuated CORT-induced apoptosis in HT22 cells. (**a**) Effects of different concentrations of PAPZ on CORT-induced HT22 cell viability determined by cell counting kit-8 (CCK-8 assay). (**b**) Fluorescein isothiocyanate (FITC)-Annexin V and propidium iodide (PI) staining followed by flow cytometry was performed to evaluate cell apoptosis of the HT22 cells. Living cells can be categorized as double negative (Q1). Early apoptotic cells can be classified as single positive or FITC-Annexin V positive (Q2). Late apoptotic cells are double positive (Q3). Dead cells can be categorized as single positive or PI positive (Q4). (**c**) The statistical analysis of total apoptosis in flow cytometry test. Data from the CORT group and PAPZ+CORT group were normalized to the control group and data are presented as mean ± SEM. * *p* < 0.05, ** *p* < 0.01, *** *p* < 0.001 represent significant differences.

The HT22 cell line is a widely used model to evaluate the pharmacological effects of potential antidepressant drugs [37,38]. To detect the effect of PAPZ on CORT-induced HT22 cells death (Figure 5), we selected 400 µM CORT, which decreased the viability of the HT22 cells by 30% (Supplementary Figure S1), as the optimal dose of the cell model to detect the effect of PAPZ on CORT-induced HT22 cells death. At these experimental conditions, the viability of the HT22 cells was significantly reduced in the CORT group when compared to the control group. Co-treatment with PAPZ increased the viability of HT22 cells in a dose-dependent manner when compared with CORT-only treated HT22 cells. The viability of HT22 cells in the PAPZ+CORT treated group was significantly higher than that in the CORT group, which indicated that PAPZ promoted the cell proliferation of HT22 cells (Figure 5a).

Flow cytometry analysis using conventional FITC-Annexin V and PI staining was performed to characterize three types of neuronal death. Total apoptosis in HT22 cells treated with CORT showed a significant increase when compared with the control group. PAPZ treatment decreased the percentage of both early and late apoptosis in HT22 cells when compared to the CORT group (Figure 5b). The statistical analysis of total apoptosis is provided in Figure 5c. The results suggested that HT22 cells were likely to undergo apoptotic rather than necrotic death when treated with CORT. Treatment with PAPZ could prevent against apoptosis induced by CORT. Our results suggested that PAPZ exerted a neuroprotective effect through inhibiting the apoptosis of HT22 cells induced by CORT.

## 4. Discussion

Given its long history usage in Asia, a reliable amount of clinical experience with TCM has been accumulated, forming a comprehensive and vital medical and cultural system. Chinese herbal medicine is the fundamental of TCM, which includes several medicinal herbs. The typical characteristics of TCM treatments are “multi-component, multi-channel, and multi-target”, which is a requirement for treating complex diseases including depression or MDD [39].

Repeated CORT injection in rodents have been considered as a reliable animal model for evaluating chronic stress depressive disorder [27]. The TST test is one of the classic experiments used to evaluate the anti-depression pharmacology of drugs and has often been used to observe the anti-depression effect of drugs by causing behavioral despair in animals [40,41]. In the present study, compared with the control group, a significant prolonged immobility time in the TST test was observed in the CORT-treated group mice. As expected, the mice treated with PAPZ+CORT exhibited a significant decrease in immobility time as compared to the CORT group (Figure 1b). PAPZ showed minor side effects on locomotor activities at the indicated dose (Figure 1c,d). Depression is often accompanied by cognitive impairment, and especially obvious learning and memory impairment [42]. In this study, the NOR test and MWM test were used to evaluate the learning and memory ability of mice. A significant increase in the DI score for new objects and new locations was shown in the PAPZ+CORT-treated group compared with the CORT-only treated group (Figure 2a,b). From the MWM results (Figure 2c), we found that the latency period of escape increased in the model group even though there was no statistical difference, whereas treatment with PAPZ significantly decreased the latency. These results demonstrated that PAPZ slightly decreased the progression of the chronic treatment of CORT-induced mild stress-mediated depressive-like behaviors as observed by the TST, NOR, and MWM tests.

Studies have shown that various pathophysiological mechanisms are involved in depression and cognitive impairment. Synaptic dysfunction and structural damage have become the focus of research. BDNF functions as an endogenous growth factor within the central nervous system [43], playing a key role in processes such as neuronal differentiation and growth, synapse formation and synaptic plasticity, and higher cognitive functions [44,45]. BDNF has also been implicated in a number of psychiatric disorders, including schizophrenia, and the development of mood disorders such as MDD and its treatment [46]. Many experiments have shown that chronic stress can down-regulate the expression of BDNF protein in the hippocampus, and this phenomenon can be effectively reversed by antidepressant therapy [47,48,49]. Neto et al. [49] found that up-regulation of neurotrophic factor expression and its signaling pathway activity could be a common pathway of antidepressant action. Therefore, we speculated that PAPZ may promote neurogenesis or change neuroplasticity by increasing the level of BDNF protein, and thus play an antidepressant role in reversing hippocampal atrophy and cell damage. According to the results of the qPCR and Western blot tests, we found that the expression of hippocampal BDNF protein in the repeated CORT injection group significantly decreased when compared with the control group. Treatment with PAPZ showed significantly increased the expression of BDNF in the hippocampal tissue when compared to the CORT group (Figure 3). Therefore, we concluded that PAPZ might exert an antidepressant role by up-regulating the expression of BDNF.

The antioxidant defense system is comprised of a series of enzymatic and non-enzymatic components. SOD is a critical antioxidant enzyme that can remove oxygen free radicals produced by the body and protect tissues from damage. Maes et al. [50] revealed that the pathogenesis of MDD might primarily or secondarily be related to oxidative stress. Antidepressant drugs function by affecting the oxidative or antioxidative systems [51], and are partly connected with their effects on the immune system [52]. Many reports have shown that disturbances in SOD activity are generally dysregulated in depressed populations. For example, the SOD activity of a red blood cell was reported to be lower in MDD patients [53]. Freitas et al. [2] reported that acute restraint stress can increase SOD activity and this effect can be abrogated by treatment with agmatine; however, no changes were observed in the unstressed animals. These disagreements in the data on SOD activity could be explained by means of variations in different drugs or a different administration period.

The results of our study showed that the SOD activity of hippocampal tissue in the CORT group mice significantly decreased, indicating that the function of the oxygen free radical defense system of depressed mice was significantly reduced, and the oxygen free radicals could not be effectively removed. PAPZ administration could reverse the trend of SOD activity. The increased SOD activity was in line with a decreased MDA level (Figure 4). Therefore, we concluded that the role of PAPZ in anti-depression could be related to the involvement of SOD and MDA. The results (Figure 5) demonstrated that PAPZ significantly attenuated CORT-induced neuroexcitotoxicity in HT22 cells, which was indicated that PAPZ could protect HT22 cells in vitro. However, the complex mechanism of PAPZ are not yet completely clear, which need to be explored in further study. In future studies, we will pay more attention to the exploration of the relevant herb of PAPZ under the guidance of the theory and practice of TCM, and clarify the role of each herb and the relationship and influences among them. 

## 5. Conclusions

In summary, a modified TCM formula (PAPZ) protected HT22 cells from CORT-induced apoptosis. PAPZ ameliorated behavioral changes induced by chronic CORT injections in the TST, NOR, and MWM tests. The essential mechanism involved in the protective effects of PAPZ in vivo included augmentation of the BDNF level and SOD activity in the hippocampal tissue. These findings provide scientific evidence for the neuroprotective and antidepressant effects of PAPZ, which suggest potential benefits of using PAPZ in the treatment or rehabilitation of MDD patients.

## Figures and Tables

**Table 1:** Chinese herbs included in PAPZ.

Scientific Name	Family Name	Source	Proportion of PAPZ
*Panax ginseng* C.A. Meyer	Araliaceae	Beijing, China	25%
*Angelica sinensis* (Oliv.) Diels	Umbelliferae	Gansu, China	25%
*Polygala tenuifolia* Willd	Polygalaceae	Shanxi, China	25%
*Ziziphi spinosae* Semen	Rhamnaceae	Hebei, China	25%

## Supplementary Materials

The following are available online at https://www.mdpi.com/2072-6643/11/4/859/s1. Figure S1: Effects of different concentrations of CORT on TH22 cell viability determined by CCK-8 assay; Table S1: Effects of different concentrations of CHFs on CORT-induced HT22 cell viability determined by CCK-8 assay.
