# Supplementary material for: The Chinese Herbal Formula PAPZ Ameliorates Behavioral Abnormalities in Depressive Mice
Source: Nutrients. 2019 Apr 16;11(4):859. doi: 10.3390/nu11040859 (PMC6521158; doi:10.3390/nu11040859)
Supplement: Supplementary file 1 [file nutrients-11-00859-s001.pdf]

Table S1. Effects of different concentrations of CHFs on CORT-induced HT22 cell viability determined by CCK-8 assay.

CHFs Code	CORT	Concentration of CHFs (mg/mL)			
		**0.5**	**5**	**10**	**50**
WXJ-17-001	74.2% ± 8.4% ###	75.3% ± 12.9%	109.7% ± 24.7% ***	133.4% ± 21.7% ***	2.1% ± 1.4% ***
WXJ-17-002	73.6% ± 7.2% ###	82.2% ± 8.9% **	114.4% ± 15.6% ***	115% ± 14.6% ***	21.6% ± 8.4% ***
WXJ-17-003	80.1% ± 16.9% ###	106.8% ± 11.8% ***	111.3% ± 26.1% ***	26.1% ± 19.0% ***	5.3% ± 1.6% ***
WXJ-17-004	75.0% ± 8.3% ###	71.7% ± 9.4%	93.7% ± 15.3% ***	105.1% ± 22.2% ***	101.1% ± 31.2% ***
WXJ-17-005	70.3% ± 6.9% ###	71.0% ± 7.0%	104.6% ± 21.9% ***	69.3% ± 20.5%	6.3% ± 0.9% ***
WXJ-17-006	70.2% ± 8.7% ###	75.2% ± 7.8%	99.7% ± 12.7% ***	104.1% ± 27.7% ***	15.9% ± 10.0% ***
WXJ-17-007	69.7% ± 10.9% ###	73.0% ± 10.9%	101.4% ± 18.5% ***	99.1% ± 32.7% ***	4.5% ± 2.1% ***
WXJ-17-008	74.0% ± 8.7% ###	81.2% ± 13.1%	96.6% ± 28.3% **	57.5% ± 31.1% *	3.9% ± 1.5% ***
WXJ-17-009	69.2% ± 7.6% ###	76.2% ± 5.2% *	87.7% ± 12.9% ***	94.4% ± 14.9% ***	5.9% ± 3.5% ***
WXJ-17-010	73.0% ± 5.4% ###	87.0% ± 5.7% ***	73.1% ± 11.2%	24.4% ± 17.4% ***	4.8% ± 1.5% ***
WXJ-17-011	75.5% ± 10.4% ###	75.3% ± 12.0%	91.9% ± 25.9% *	86.2% ± 34.2%	1.9% ± 0.9% ***
WXJ-17-012	76.1% ± 11.2% ###	80.8% ± 23.2%	50.1% ± 22% ***	14.6% ± 5.1% ***	4.9% ± 1.0% ***
WXJ-17-013	72.0% ± 6.4% ###	76.7% ± 12.1%	77.7% ± 15.6%	24.7% ± 27.9% ***	2.0% ± 0.6% ***
WXJ-17-014	72.0% ± 7.5% ###	64.4% ± 8.9% **	77.0% ± 17.0%	70.6% ± 25.1%	2.3% ± 1.2% ***
WXJ-17-015	69.2% ± 7.6% ###	60.7% ± 15.8% *	56.0% ± 17.8% **	19.4% ± 9.5% ***	2.7% ± 1.3% ***
WXJ-17-016	70.5% ± 9.7% ###	64.4% ± 11.2%	33.9% ± 6.6% ***	10.5% ± 4.1% ***	3.8% ± 1.2% ***
WXJ-17-017	78.2% ± 11.4% ###	81.2% ± 8.2%	90.9% ± 14.1% **	24.6% ± 10.0% ***	2.1% ± 0.7% ***
WXJ-17-018	76.4% ± 12.8% ###	79.6% ± 10.7%	84.2% ± 11.2%	50.8% ± 39.1% **	1.2% ± 0.7% ***
WXJ-17-019	78.6% ± 11.2% ###	79.4% ± 11.1%	81.4% ± 10.5%	78.4% ± 14.1%	37.5% ± 18.6% ***
WXJ-17-020	75.4% ± 12.2% ###	71.3% ± 11.0%	75.9% ± 12.3%	71.0% ± 16.1%	67.1% ± 23.8%
WXJ-17-021	75.5% ± 10.3% ###	69.8% ± 9.2%	71.4% ± 12.8%	66.7% ± 15.1% *	43.8% ± 13.9% ***
WXJ-17-022	78.2% ± 11.4% ###	81.2% ± 8.2%	90.9% ± 14.1% **	24.6% ± 10.0% ***	2.1% ± 0.7% ***

CHFs, Chinese herbal formulas; CORT, corticosterone; cell counting kit-8, CCK-8; ###*p* <0.001, compared with the control group (the cell viability of control group was 100%); **p* <0.05, ***p* <0.01, ****p* <0.001, compared with the CORT group.

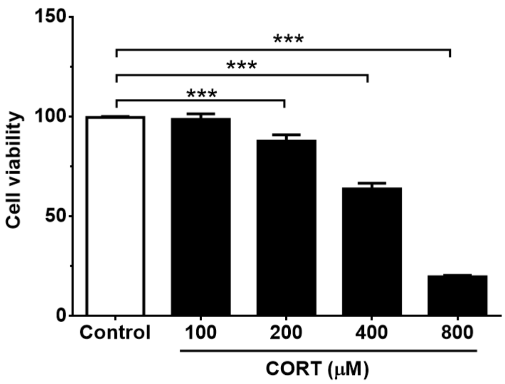

Figure S1. Effects of different concentrations of corticosterone (CORT) on TH22 cell viability determined by cell counting kit-8 assay. ***p <0.001, compared with the control group.
